# Analysis of ParB-centromere interactions by multiplex SPR imaging reveals specific patterns for binding ParB in six centromeres of Burkholderiales chromosomes and plasmids

**DOI:** 10.1371/journal.pone.0177056

**Published:** 2017-05-31

**Authors:** Flavien Pillet, Fanny Marie Passot, Franck Pasta, Véronique Anton Leberre, Jean-Yves Bouet

**Affiliations:** 1 Ingénierie des Systèmes Biologiques et des Procédés INRA UMR792, Institut National de la Recherche Agronomique, Institut National des Sciences Appliquées, Toulouse, France; 2 Laboratoire d’Ingénierie des Systèmes Biologiques et des Procédés CNRS UMR5504, Centre National de la Recherche Scientifique, Institut National des Sciences Appliquées, Université de Toulouse, Toulouse, France; 3 Laboratoire de Microbiologie et Génétique Moléculaires, Centre de Biologie Intégrative, Centre National de la Recherche Scientifique, Université Paul Sabatier, Toulouse, France; University of Oklahoma, UNITED STATES

## Abstract

Bacterial centromeres–also called *parS*, are cis-acting DNA sequences which, together with the proteins ParA and ParB, are involved in the segregation of chromosomes and plasmids. The specific binding of ParB to *parS* nucleates the assembly of a large ParB/DNA complex from which ParA—the motor protein, segregates the sister replicons. Closely related families of partition systems, called Bsr, were identified on the chromosomes and large plasmids of the multi-chromosomal bacterium *Burkholderia cenocepacia* and other species from the order Burkholeriales. The centromeres of the Bsr partition families are 16 bp palindromes, displaying similar base compositions, notably a central CG dinucleotide. Despite centromeres bind the cognate ParB with a narrow specificity, weak ParB-*parS* non cognate interactions were nevertheless detected between few Bsr partition systems of replicons not belonging to the same genome. These observations suggested that Bsr partition systems could have a common ancestry but that evolution mostly erased the possibilities of cross-reactions between them, in particular to prevent replicon incompatibility. To detect novel similarities between Bsr partition systems, we have analyzed the binding of six Bsr *parS* sequences and a wide collection of modified derivatives, to their cognate ParB. The study was carried out by Surface Plasmon Resonance imaging (SPRi) mulitplex analysis enabling a systematic survey of each nucleotide position within the centromere. We found that in each *parS* some positions could be changed while maintaining binding to ParB. Each centromere displays its own pattern of changes, but some positions are shared more or less widely. In addition from these changes we could speculate evolutionary links between these centromeres.

## Introduction

Partition (Par) systems were discovered on low-copy number plasmids to whom they ensure active segregation and thus stability over generations. They consist of three elements: a NTPase, called ParA, a DNA-binding protein called ParB, and a cis-acting sequence—the target of ParB, also referred to as centromere or *parS* site. *ParS* is generally a small repeated sequence, clustered in plasmids [1] while dispersed in chromosomes in the origin domain [2]. Par B binds to *parS* forming a nucleo-protein complex with which ParA interacts to lead the segregation of daughter replicons [3]. Three main types of Par systems are defined according to the nature of the NTPase: Walker A—P loop ATPases (type I), actin-like ATPases (type II) and tubulin-like GTPases (type III) [4–6]. While in types II and III the NTPase polymerization is involved in driving plasmids apart, the segregation mechanism remains unclear in type I, the most common Par system of plasmids and the only one found on chromosomes.

In type I, the basic *parS* repeat generally consists of two inverted repeats (IR), either forming a palindrome, or with a spacing of a few base pairs between the two IRs. ParB proteins are functional dimers [7], so that each monomer of the dimer could interact with one IR of *parS*, as proposed for the ParB protein of the F plasmid [8,9]. The specific binding of ParB to *parS* nucleates the formation of an extended nucleo-protein zone called “partition complex” wherein ParB binds DNA without sequence specificity [10,11].

Chromosomal Par systems do not provide all the driving force for segregating the bulk of sister chromosomes, but they control the segregation of new-born origins of replication [12–14] and, when transplanted on plasmids deleted of their own Par systems, they stabilize these plasmids [15–18]. Chromosomal Par systems are therefore true partitioning machines, even though this function does not appear clearly in several species because it is limited to certain physiological states [17,19], or linked to major functions of the cell-cycle like cytokinesis [20], initiation of replication [12,21,22] and DNA condensation [23–25].

The phylogenetic groups of Par systems correlate with the lineages of replicons carrying them [16,18]. For example the Par systems identified so far on chromosomes, whatever the species, are highly homologous each other and share the same 16 bp *parS* sequence—TGTTTCACGTGAAACA, initially found in *Bacillus subtilis* [15]. This small palindrome is therefore a faithful marker of the “chromosome family”. More-over, in the few bacterial genomes possessing more than one chromosome, the longest chromosome also displays this *parS* site, suggesting it originates from the same group as single chromosomes. In contrast, secondary chromosomes of multi-chromosomal genomes have Par systems more related to those of plasmids and, like the Par systems of plasmids, they identify specific lineages of replicons [17, 26].

*Burkholderia cenocepacia* is a multi-chromosomal proteobacterium from soils and roots, identified as an aggravating agent in cystic fibrosis [27]. J2315, one of the most virulent strain of *B*. *cenocepacia* [28], has three chromosomes of 3.9, 3.2 and 0.9 Mb (c1, c2 and c3 respectively), and one plasmid of 93 kb (pBC). A partition (Par) system was found on each replicon of J2315 [18]. Par systems of c2, c3 and pBC, together with those of certain large plasmids of other Burkholderiales, are phylogenetically related and identify sub-families of Par systems called Bsr (Burkholderiales secondary replicon) [26]. Bsr Par systems display as *parS* site a palindrome of sixteen base-pairs, with a T-rich 5’-end, and a six base-pairs core composed of 2C, 2G, 1A, 1T which includes a central CG dinucleotide. Surprisingly, these *parS* sites resemble the chromosomal *parS* described above which is also present on c1 of *B*. *cenocepacia*.

To avoid incompatibility among the four replicons, each *parS* of *B*. *cenocepacia* should not bind ParB of another replicon. Accordingly, the *parS* motifs are replicon-specific and, despite their similarities, they do not bind non-cognate ParBs *in vivo* or *in vitro* [18,26]. We proposed that the Par systems of *B*. *cenocepacia* have a common ancestry and evolved for their compatibility in *B*. *cenocepacia*, erasing any trace of cross reaction. This proposal was supported also by the converse fact that binding was detected between ParB c3 and *parS* of plasmids from other species, *i*.*e*. between Par systems of replicons evolving without selection pressure for compatibility. Thus, the binding of *parS* to ParB, crucial for the segregation process, looks also essential for discriminating between replicons in the multi-chromosomal genomes of the Burkholderiales. The same conclusion was drawn recently for the multipartite *Rhizobium leguminosarum* genome [29].

**Fig 1:**
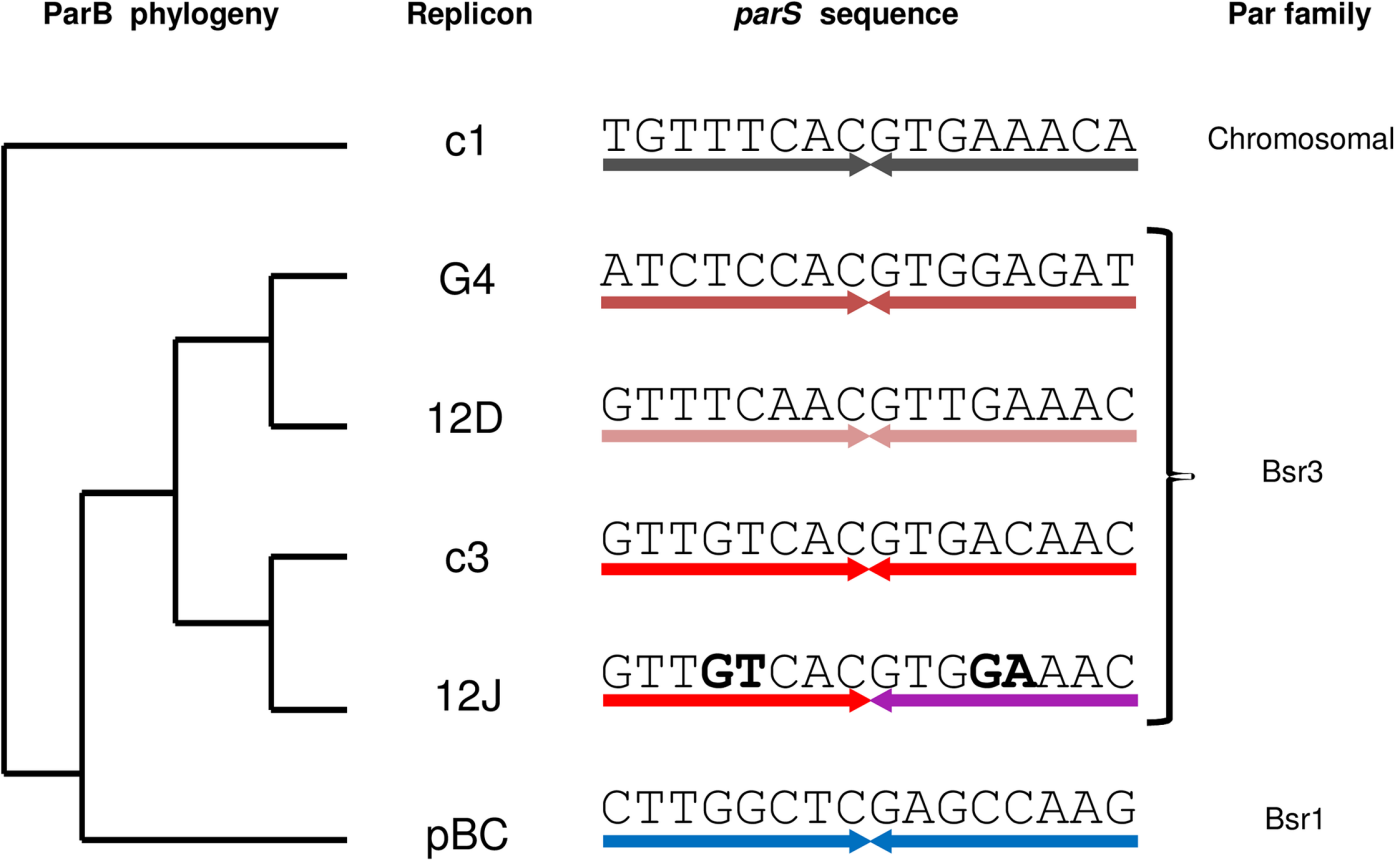
ParB phylogeny and *parS* sequences of the Par systems used in this study. ParB Phylogeny was obtained using the multiple sequence alignment webPRANK of the European Bioinformatic Institute (http://www.ebi.ac.uk/goldman-srv/webprank/). The Par systems are from the following replicons: c1, chromosome 1 of *Burkholderia cenocepacia* J2315; G4, plasmid 2 of *Burkholderia vietnamiensis* G4; 12D, plasmid 1 of *Ralstonia pickettii* 12D; c3, chromosome 3 of *B*. *cenocepacia* J2315; 12J, plasmid integrated in the chromosome of *Ralstonia pickettii* 12J; pBC, plasmid pBC of *B*. *cenocepacia* J2315. Families correspond to those previously established based on a wider collection of Par systems. All *parS* sequences form a perfect palindrome (indicated by the inverted arrows with specific colours for specific sequences), except *parS*12J which displays non complementary bases (boldface).

We previously analyzed the effect of some nucleotide changes in *parS*c1 and *parS*c3 on the binding to ParB, either *in vitro* by Electrophoretic Mobility Shift assay (EMSA) and *in vivo* by plasmid incompatibility [26]. Few nucleotides could be changed in *parS*c1 and *parS*c3 while the binding to ParB was maintained. To get more insights on the specificities for binding ParB of the Burkholderiales centromeres we have expanded here the study to other nucleotide changes and to other Burkholderiales Par systems. To do this, we have used the Surface Plasmon Resonance imaging (SPRi) multiplex technology, which allows hundreds of DNA probes to be tested at the same time. Systematic nucleotide changes were analyzed in the *parS* sequences of six Par systems: c1 (chromosome 1 of *Burkholderia cenocepacia* J2315), c3 (chromosome 3 of *B*. *cenocepacia* J2315), pBC (the plasmid of *B*.*cenocepacia* J2315), 12D (the plasmid 1 of *Ralstonia pickettii* 12D), 12J (a plasmid integrated in the chromosome of *R*. *pickettii* 12J and the plasmid 2 of *R*. *pickettii* 12D) and G4 (plasmid 2 of *Burkholderia vietnamiensis* G4). All are from Burkholderiales replicons and, as already mentioned, the *parS* sequences of these systems resemble each other. 12J, G4 and 12D belong to the same family, Bsr3, which is that of c3 ([26] and Fig 1) and their ParBs have 54, 45 and 40 percent identity respectively with ParBc3. On the other hand, pBC and c1 belong to other Par families (Fig 1) and their ParBs have respectively 31 and 26 percent identity with ParB c3. It seemed therefore interesting to analyze this panel, in order to find if patterns of permissive positions for binding ParB in each *parS* correlate the relationship and could infer some evolving link between these Par systems.

## Material and methods

### Sources of the Burkholderiales *parB* genes

*ParB* genes from *Burkholderia cenocepacia* J2315 (DSMZ), *Burkholderia vietnamiensis* G4 (J. Tiedje, Michigan State University), *Ralstonia pickettii* 12D and *Ralstonia pickettii* 12J (T. Marsh, Michigan State University) were amplified by PCR (Phusion High-Fidelity DNA Polymerase, Thermofisher Scientific) on genomic DNA extracted from these species.

### ParB cloning and extract preparation

ParB extracts were prepared as described previously [26]. Briefly, wild-type *parB* genes cloned from start to stop codon with a consensus Shine-Dalgarno sequence were inserted under the control of the arabinose inducible *pBAD* promoter into the pDAG127 vector [30] with a deletion of the *sopA* gene by NheI/HindIII or EcoRI/HindIII digestion. Recombinant plasmids were electroporated into the *Escherichia coli* strain DLT812 [31]. Cells grown in LB medium at 37°C to an optical density at 600 nm (OD_600_) of 0.25 were induced with 0.1% arabinose (final concentration), incubated for 4 h at 37°C, and chilled on ice for 10 min. Subsequent steps were carried out at 4°C. Cells were centrifuged, washed with TNE (50 mM Tris-HCl [pH 7.5], 50 mM NaCl, 1 mM EDTA) and resuspended in TNE at an OD_600_ of 300. Lysozyme was added at 400 μg/ml; cells were then incubated for 5 min, frozen with liquid nitrogen, and thawed on ice. Lysis buffer (TNE, 660 mM NaCl, 4.5 mM dithiothreitol) was added to bring the OD_600_ to 200. The mixture was incubated for 10 min on ice, and the cells were lysed by sonication. The lysate was centrifuged for 15 min at 10,600 × *g* and the supernatant centrifuged at 20,800 × *g*. The final supernatant was frozen with liquid nitrogen and stored at—20°C. Total protein concentrations were measured using a Bradford protein assay (Bio-Rad). Samples containing 3.5 μg of protein were electrophoresed in 4 to 12% poly-acrylamide bis-tris denaturing gels (NuPage; Invitrogen) at a constant voltage of 200 V for 55 min and then stained with a Coomassie blue derivative (InstantBlue Expedeon). Lanes containing no protein or protein from cells without ParB were also included on gels, and stained gel images captured with a gel-imager (Syngene G box) as.tif files were scanned and processed using Multi Gauge (Fuji) to quantitate roughly ParB as detailed previously [26]. The concentrations of ParB were estimated to range between 0.4 μg/ml (ParBpBC) and 2.8 μg/ml (ParBc1).

### *ParS* probes and spotting conditions

**Fig 2:**
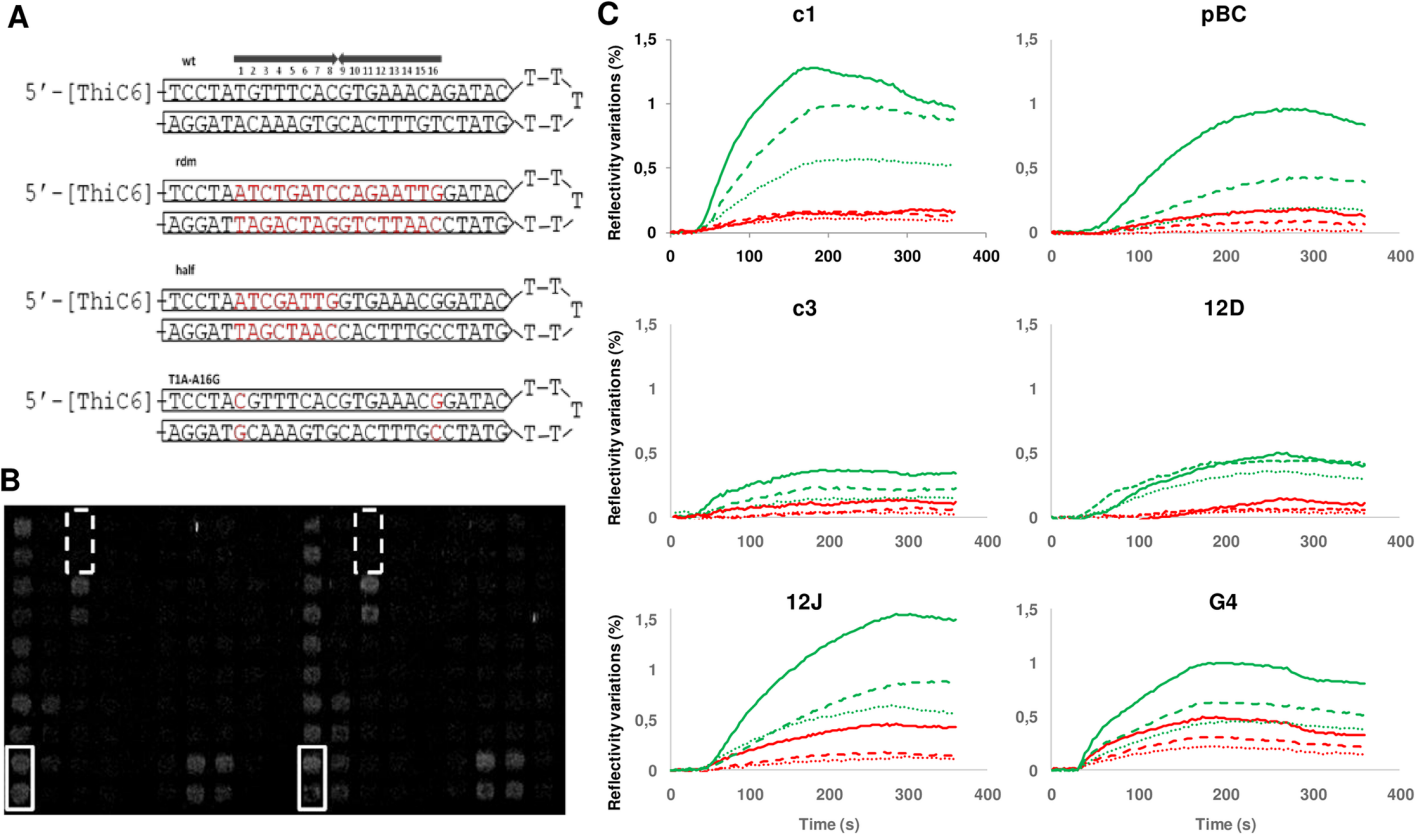
Experimental design. **(A)** Structures of the 5’-thiol-labelled 57-mer single strand oligonucleotides carrying carrying *parS*, exemplified for *parS*c1. The *parS* sequence forms by the annealing of two inverted repeats of 26 bases (arrowed boxes). The upper structure shows the wild-type (wt) 16 bp palindrome of *parS*c1 by two arrowed black lines and with bp positions numbered. Three sequences modified from *parS*c1 are indicated below: randomly modified (rdm), one arm modified (half), and the double symmetric change T1C-A16G, (changed sequence is written in red) **(B)** Typical SPRi image captured at the end of injection (240 sec) of 10μg/ml of crude extract enriched in ParB c1. Each DNA probes was distributed in duplicate in two different localizations, as shown by white rectangles with *parS* wild type sequence (c1 wt) and white dotted rectangles with randomized sequence (c1 rdm). **(C)** Examples of kinetics curves for the six ParB-*parS* systems. For each ParB, the reflectivity variations according to time were observed with the wild-type *parS* (green curves) and the random *parS* (red curves). Various concentrations of crude extracts were injected: 10 (dotted lines), 20 (dashed lines) 40 μg/ml (full lines) for c1; 20 (dotted lines), 40 (dashed lines) and 80 μg/ml (full lines) for pBC, c3, 12D, 12J and G4.

All *parS* probes were designed as previously described [9], using 57-mer oligonucleotides modified at their 5’-ends with a thiol function (Sigma Aldrich, France). They consist of two inverted repeats of 26 bases–including the 16 bases *parS* or derivative sequences—separated by a five base spacer (see Fig 2A). Each oligonucleotide, resuspended at 3 μM in 3X SSC buffer containing 450 mM NaCl and 45 mM sodium citrate pH 7 (Sigma Aldrich, France) were spotted (25μM) on SPRi-Biochips covered with a 50 nm gold film using a ChipWriterPro contact spotter (Biorad) with solid pin SSP015 from Arrayit Corporation. For each probe, four spots were deposited in tandem at two different locations on the prism (Fig 2B).

### SPRi assays

SPRi experiments were performed using a SPRi-Plex system (GenOptics HORIBA-scientific, France) as described [32] using a running buffer (20 mM HEPES pH 7.4, 100 mM KCl, 0.1% bovine serum albumin—BSA, 50μg.ml^−1^ sonicated salmon sperm DNA) at a flow rate of 50μl.min^−1^. 200μl of ParB over-expressing crude cell extracts were injected during 240 s on the prism surface saturated with Denhardt solution (Sigma). The variation of reflectivity (VR) vs. time for each spot was analysed with Genoptics software SPRiAnalysis1.2.1. Mean values and standard deviations were directly calculated with this software from four independent spots. Between each injection, the regeneration of the prism surface was performed with NaOH 100 mM. The level of significance relative to the negative control (a sequence modified randomly relative to *parS* and indicated “rdm”) was determined by the student T test in excel software. Levels of significance are indicated in the “Sign.” columns of Figs A-G in S1 File as follows: * (P value < 0.05), ** (P value < 0.01), *** (P value <0.001). In addition a crude extract non over-producing ParB (referred to as ParB- extract) was also injected. For most probes the values obtained with this extract allow to discard the possibility that *E*. *coli* proteins contribute to the reflectivity. For the few probes that seemed to interact with proteins of the ParB- extract, we considered by default the VR obtained with ParB as non-significant—*e*.*g*. probes S160 and S161 (Fig F in S1 File), and probe S142 (Fig G in S1 File).

## Results

### Setting up the SPRi conditions to determine ParB/*parS* interactions

The goal of this study was to analyse the interaction of six ParB proteins originating from six *Burkholderiales* replicons (chromosome, secondary chromosome or plasmid) with their cognate *parS* and many derivatives, in order to get insights on the specificities and similarities of these Par systems. Surface Plasmon Resonance imaging (SPRi) is adapted for such a wide analysis. Oligonucleotides allowing the formation of *parS* or derived sequences (exemplified in Fig 2A) are spotted on the same prism. ParB is injected and the variation of reflectivity was measured at each oligonuclotide spot (Fig 2B). Crude *Escherichia coli* extracts over-expressing one ParB were used in this study, as they are at least as sensitive as purified ParB proteins in detecting DNA probes in SPRi assays [9]. Three protein concentrations were used: 10, 20 and 40 μg/ml for ParBc1; 20, 40 and 80 μg/ml for the 5 other ParBs. Upon injection of ParB, the variation of reflectivity (VR) for each ParB increases with time up to a maximum value, indicating that equilibrium between association and dissociation is reached. For each ParB, the VR obtained with the cognate *parS* is above that obtained with the randomly changed *parS* (Fig 2C, compare green and red curves for the same protein concentrations) indicating that the specific binding for cognate *parS* is detected. Increasing the protein concentrations leads to globally increased curves of VR, suggesting the used concentrations are not saturating, and thus valid to determine ParB-*parS* interactions. In some cases the VR decreases before the end of injection (240 s), which nevertheless suggests a mass transport due to excess proteins. This is particularly noticeable for ParBc1 at the highest concentration (Fig 2C). For ParB12D there is a time delay for detecting the increase of VR, which also occurs at the highest protein concentration. To correlate thereafter the VR and the interaction of ParBs with modified *parSs*, we thus kept the VR obtained with the smallest (10 μg/ml for ParBc1) or the intermediate (40 μg/ml for other ParBs) protein concentrations. For each ParB / *parS* interaction tested, we defined a binding score (BS) by taking the VR value at the end of injection. Many probes with four or more changes on the full *parS* length have been tested, giving mostly VR close to non specific, so that we will not discuss these results in details. They indicate roughly that *parS* sequences cannot be changed drastically to maintain a well detected ParB/*parS* interaction by SPRi. The results that we present and discuss are those relative to the probes designed to (i) characterize the binding of ParB to one arm of *parS* and (ii) determine the importance of each position in *parS* for binding ParB.

The sequences of all tested probes, the BS obtained with extracts over-producing ParB (ParB+) and extracts non-producing ParB (ParB-), the standard deviations and the significance of the values are shown in S1 File.

### Mutating one arm of the palindromic *parSs*

With the exception of *parS*-12J, the studied *parSs* are perfect palindromes of 16 bp (Fig 1). They consist of two contiguous inverted repeats (IR) of 8 bp, referred to as *parS* arms. ParBs of the present study have a helix-turn-helix (HTH) domain previously identified [17, 26], so that each monomer of the ParB dimer could interact with one arm of *parS*, as it is the case for the ParB protein of the F plasmid [8, 9]. Some probes designed with one nucleotide change in just one arm of *parS*-c1, -c3, and -pBC, gave high BS, similar to those of the wt controls (T1G, T1A, T3C, T3A, T5A in *parSc1*;G1T, G1C, C6T in *parS*c3; C1G, C6A, C6T, T7G in *parS*pBC–Figs A-C in S1 File). However these probes do not allow determine if the remaining normal arm is responsible for the high BS or if the nucleotide change impairs poorly the interaction. To convincingly determine if one arm maintains binding to ParB we designed oligonucleotide probes, called “half-*parS*”, with one arm randomly changed (Figs A-G in S1 File, and Fig 2A for c1).

**Fig 3:**
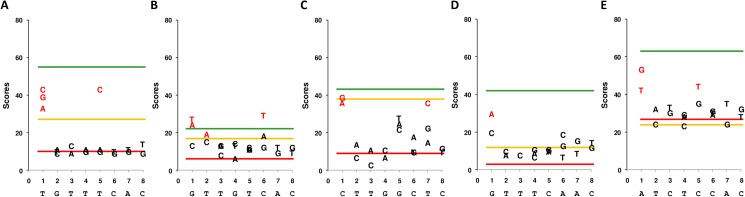
Binding scores for palindromic *parS* and derivatives. (A) ParB c1 *B*. *cenocepacia;* (B) ParB c3 *B*. *cenocepacia;* (C) ParB plasmid pBC *B*. *cenocepacia*; (D) ParB plasmid 1 *Ralstonia picketti* 12D; (E) ParB plasmid 2 *Burkholderia vietnamiensis* G4. Binding scores correspond to 100-fold the VR value at the end of the SPRi injection. The average scores for the wild-type (wt) and random (rdm) sequence are represented as lines, green and red respectively. Average scores for the half-*parS* sequences are shown as orange lines. For double symmetric substitutions, the score is directly pointed on the graph as the base substituting the wild-type one indicated below on the x-axis. Changes are indicated for one arm only but correspond to symmetrical changes (*e*.*g* the change T1C in *parS*c1 means T1C and A16G). For each change statistical analysis of the BS were performed; the substitutions displaying a significantly different BS relative to that of non specific DNA are wrote in red.

For c1, c3, and pBC (Fig 3A, 3B and 3C) the BSs of half-*parS* (orange lines) are intermediate *i*.*e*. below the specific BS (wt, green lines) and significantly above the non-specific BS (rdm, red lines). Thus, in these cases one arm of *parS* retains specific binding for ParB, as described the centromere of the F plasmid—which is composed of two inverted repeats separated by two bases [9]. Disparities nevertheless appear between the BS induced by these three half-*parS*s: that of pBC allows a very high BS, just below the wt *parS* pBC, while half-*parS*c1 and half-*parS*c3 generate weaker BS relative to their wt *parS*.

For 12D and G4, the BSs of half-*parSs* are just above and at the non-specific value respectively (Fig 3D and 3E). We cannot formally exclude that SPRi could not discriminate between reduced and non-specific interactions due to a lack of sensitivity. But this seems unlikely because with the same ParB extracts (12D and G4) tested on other derivatives of *parS*G4, SPRi was able to reproducibly measure weak BSs, just above the non-specific value (Fig 3D and 3E). If there is a lack of sensitivity of the SPRi, it does not question the fact that the interaction of ParB12D and ParBG4 with half-*parS*G4 is weak, close to the interaction observed with non-specific DNA.

### Double symmetrical mutations in palindromic *parS*s

To determine the relative importance of each nucleotide at a given position of the five palindromic centromeres we designed probes mutated at each pair of symmetrical positions (*i*.*e* 1–16, 2–15, 3–14, 4–13, 5–12, 6–11, 7–10 and 8–9) by two or three complementary substitutions. These probes were assayed by SPRi (Fig 3A–3E).

For the five palindromic *parS* the distal position (1–16) allows the largest amount of substitutions while maintaining a BS well above that of the non-specific DNA, even reaching or exceeding the BS of the wt *parS* in the case of c3. Some changes, internal to the *parSs*, also maintain a BS well above the non-specific. This is notably the case at positions (2–15) and (6–11) in *parS*c3, (5–12) in *parS*c1, (7–10) in *parS*pBC and (5–12) in *parS*G4. For these internal positions the highest BSs correspond in general to transitions, *e*.*g*. T5C in *parS*c1, C5T in *parS*G4, C6T in *parS*c3 and T7C in *parS*pBC. The highest BS for internal transversions is T2A in *parS*c3.

Interestingly, in *parS*c1 the BS is completely dropped to basic by all changes (Fig 3A), with the exceptions of positions (1–16) and (5–12) already mentionned. Such a narrow specificity of each position is less obvious in the other *parSs*, notably *parS*c3 (Fig 3B).

### Changes in the non palindromic *parS*12J

**Fig 4:**
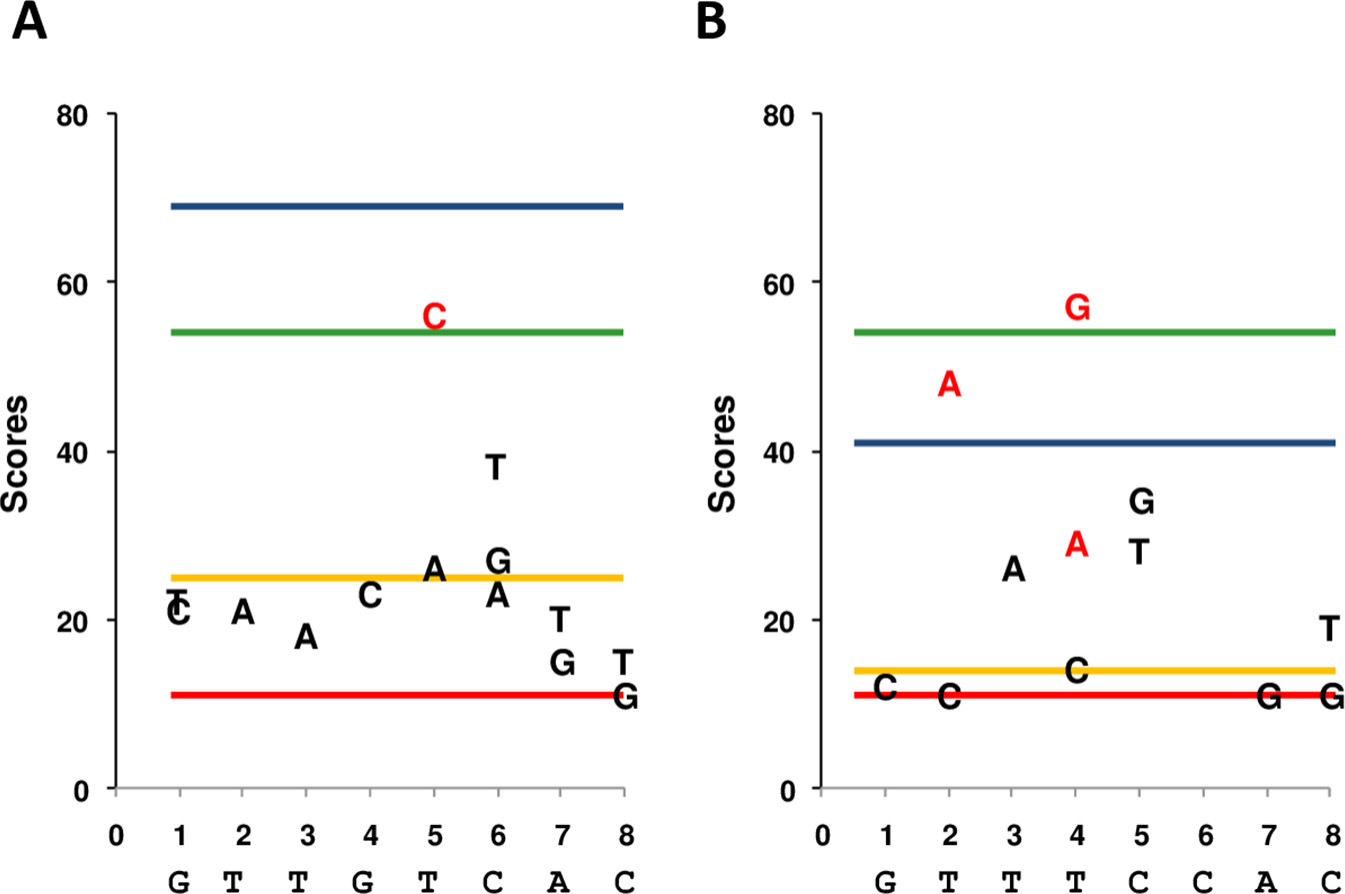
Binding scores of ParB12 J for *parS*c3 non-*parS*c3 sequences and derivatives. Blue lines correspond to the BS of the palindromic sequence *parS*c3 (A) and non *parS*c3 (B). Legend is the same as in Fig 3.

The sequence *parS*12J is repeated five times upstream the corresponding *parAB* operon on a plasmid integrated into the chromosome of *Ralstonia pickettii* 12J [26]. The arms of *parS*12J are not strictly complementary, one being identical to *parS*c3 and the second differing at two positions (Fig 1); they will be referred to as “*parS*c3” and “non-*parS*c3” arms, respectively. The interaction of ParB12J with *parS*12J and its derivative probes were assayed by SPRi (Fig 4). The reference BSs were 54 for the cognate *parS* 12J (wt, green line in Fig 4A and 4B) and 11 for the non-specific DNA (rdm, red lines in Fig 4A and 4B). The BS of ParB12J for “*parS*c3” and “non-*parS*c3” full palindromes are high (69 and 41 respectively; Fig 4A and 4B blue lines). Thus ParB12J has the capability to bind palindromic *parSs*, and the *parS*c3 palindrome appears a better substrate than the “non *parS*c3” palindrome but also better than its cognate *parS*12J. We therefore tested the interaction of ParB12J with two “half-*parSs*” sequences keeping either the “*parS*c3” or the “non-*parSc3”* arm. They displayed a BS of 25 and 14 respectively (orange lines; Fig 4A and 4B), confirming that *parS*c3 is a better substrate than non-*parS*c3, but mainly suggesting that both arms of *parS* are important for the binding ParB12J, as observed notably for ParB12D and ParBG4.

We then analyzed the effect on the BS of double symmetric changes introduced in full palindromes, either “*parS*c3” or “non-*parS*c3” (Fig 4A and 4B). In both palindromes changes at the distal position (1–16) strongly impair ParB binding. This result indicates that for ParB12J the distal position of the palindrome is important for the specificity of interaction, in contrast to all the other *parSs*. Few internal positions accept changes while maintaining a significant BS with ParB12J: position (5–12) in the “*parS*c3” palindrome; positions (2–15) and (4–13) in the “non-*parS*c3” palindrome. Interestingly, in the “non-*parSc3*” palindrome the best accepted changes are transversions: T2A and T4G (Fig 4B).

## Discussion

In this study we have investigated by an SPRi approach, what nucleotides are involved in the specific ParB/*parS* interaction for six Par systems: those of chromosome 1 (c1), chromosome 3 (c3), and the plasmid (pBC) of *B*. *cenocepacia* J2315, and those of three plasmids related to c3: plasmid 2 of *Burkholderia vietnamiensis* G4 (G4), plasmid 1 of *Ralstonia pickettii* 12D (12D) and the plasmid integrated in the chromosome of *Ralstonia pickettii* 12J (12J). The six *parS*s are similar, consisting of a 16 bp sequence—perfectly palindromic in most cases, and displaying a central CG dinucleotide. It is therefore interesting to analyze if similar *parS*s display similar nucleotide specificity.

### Validation of the SPRi technology by comparing to previous techniques

The SPRi multiplex technology allows the analysis of multiple ParB/*parS* interactions simultaneously. This is an advantage over the techniques that we previously used in the same goal, *i*.*e*. electrophoretic mobility shift assay (EMSA) and an *in vivo* test of incompatibility [26]. Briefly, for the *in vivo* test, *B*. *cenocepacia* was transformed with plasmids carrying extra-*parS* sequences; a growth delay of transformants indicated the extra-*parS* bind ParB while a normal growth indicated the extra-*parS* is impaired for binding ParB. Interestingly, the interaction with ParB of some *parS*c1, *parS*c3 and *parS*12J derivatives tested here by SPRi, was also tested by incompatibility and (or) EMSA, allowing to discuss the reliability of SPRi.

It appears that the three techniques (*in vivo* growth delay, EMSA, and SPRi) give their highest responses for the wild-type *parSs*. In addition, all the mutations that maintained a delayed growth *in vivo*, display a high SPRi BS. It is the case for single-arm mutations T1G, T1A of *parS*c1, and G1T, G1C, C6T of *parS*c3 (Figures A-B in S1 File, and [26]). It is also the case for the double symmetrical mutations T1G-A16C and T5C-A12G of *parS*c1 (Fig 3A, Figure A in S1 File, and [26]). Conversely all changes leading to a low BS did not delay growth *in vivo*: double symmetrical A7T-T10A in *parS*c1; T5G-A12C and A7T-T10A in *parS*c3 (Fig 3A and 3B; Figures A- B in S1 File, and [26]). Also, we found that the changes that maintain a relatively high BS in this study, led to a band shift in EMSA: *e*.*g* single-arm T1G and T1A in *parS*c1; double symmetrical T1G-A16C, T5C-A12G in *parS*c1, and double symmetrical C6T-G11A in *parS*c3.

We nevertheless observe that few *parS* derivatives that maintain a relatively high BS did not delay growth by incompatibility, *e*.*g* single arm mutations T3C, T3A, T4A and T5A, in *parS*c1 (Figure A in S1 File and [26]). However, these derivatives are subtly modified *parSs* (one nucleotide change on one arm), so that the detection of their interaction with ParB could be particularly dependent on the technique used. For example *in vivo*, any binding defect of the extra-*parS* should be aggravated by its competition with the genomic *parS*, rendering the *in vi*v*o* approach more stringent but less suitable than SPRi to reveal weak interactions between ParBs and modified *parS*.

Alltogether, these data suggest that, for most *parS*, especially those wild-type and their multi-mutated derivatives, SPRi compares with the other techniques. Besides, it was established for the ParB protein of the F plasmid that a two-fold reduction of the reflectivity–measured by SPRi for a half centromere site—corresponded to a 100 fold loss of affinity [9]. Certainly this observation indicates that the reflectivity is not proportional to the affinity; on the other hand it suggests that most ParB/*parS* interactions, even those lessened compared to the wt, are readily detected by SPRi. Thus SPRi is a valuable tool for the rough and wide detection of ParB/*parS* interactions.

### Influence of the symmetrical structure of *parS* for binding ParB

ParBc1, ParBc3 and ParBpBC display for their half-*parS* a BS significantly above the negative control value: 49%, 77% and 88% respectively of the BS for wild-type *parS*. This behavior is shared by the ParB protein of the F plasmid [9]; and ParB2 of *Vibrio cholerae* was found to bind a non centromeric site provided this site contains a half-*parS* sequence [33]. This suggests that for many ParBs the initial interaction may occur at one arm of *parS* only. For ParBpBC the reduction of BS is low (12%) which might further suggests that once a monomer has interacted with the normal arm, the second monomer binds DNA whatever the sequence. At the opposite, the BS for half-*parS* was severely impaired for ParB12D and dropped to basic for ParBG4. In the latter case the binding of each HTH to both *parS* arms seems necessary for the stable interaction of ParB.

ParB12J, whose natural *parS* is not symmetrical—one arm like *parS*c3 and one arm “non *parS*c3” differing at two positions, also displays the capability to bind just one arm. The *parSc3* arm is clearly the favorite (Fig 4A and 4B orange lines). More interestingly, we found that ParB12J interacts with both palindromes corresponding to the perfect inverted repeat of each arm of *parS*12J, i.e. *parS*c3 and “non *parS*c3” palindromes (Fig 4A and 4B blue lines). The BS of ParB12J was even higher with *parS*c3 than with its own *parS*12J centromere. However, the non symmetrical structure of *parS*12J is repeated identically on DNA and maintained in two replicons (in *Ralstonia pickettii* 12J on a plasmid integrated into the chromosome, and in *R*.*pickettii* 12D on a free plasmid). The conservation of this asymmetry suggests that a specific adaption of ParB to this centromere occured, but certainly not for optimizing its binding. The formation of a partition complex depends on the affinity of ParB for its minimal *parS* target but also on the number of repeats of this target, the further spreading of ParB etc… Improving the function of a Par system might sometimes be achieved through reducing the ParB/*parS* affinity.

### Determination of the positions tolerant to changes

Position (1–16) of *parS*c1, c3, pBC, 12D and G4 undergoes nucleotide changes while maintaining the interaction with the cognate ParB. This has already been reported [9,26,34] and suggests that the distal position has less importance for the specific binding of ParB. An exception appears: ParB12J, whose binding to the palindromes *parS*c3 and “non-*parS*c3” is abolished when they are doubly modified at this position. If, as discussed above, ParB specifically adapted to a non-symmetrical *parS*, this adaptation might, on the other hand, limit the spectrum of the *parS* modifications maintaining an interaction with ParB. In the context of “not natural” centromeres (*i*.*e*. palindromic in this case), the distal position (1–16) could be more crucial for the binding.

In most *parS*s we found some internal positions also permissive to changes, like position (5–12) in *parS*c1, *parS*G4 and *parS*c3 (in the latter case for binding ParB12J), position (6–11) in *parS*c3 for binding ParBc3, and position (7–10) in *parS*pBC (Figs 3 and 4). It is also the case of the position (2–15) in *parS*c3 for binding ParBc3, and in the “non-*parSc3*” palindrome for binding ParB12J. Interestingly in both cases the transversion T2A is the best tolerated change, which is atypical—we found mostly transitions in the less destabilizing changes, and strengthens the kinship previously proposed between 12J and c3. The patterns of permissive nucleotide changes thus appear partly shared by few *parS* and partly specific.

### The specific case of *parS*c1

*ParS*c1 appears the less permissive centromere to nucleotide changes: apart those distal (1–16) and the transition at (5–12), all symmetrical changes abolished the interaction (Fig 3A). This narrow specificity for binding ParB could be linked to the multi-chromosomal environment of *Burkholderia*: a nucleotide change allowing ParB of another chromosome to bind *parS* of the major chromosome would be detrimental for the genome stability. As *parS*c1 has the canonical sequence of all *parSs* of single-chromosome genomes, it is interesting to determine if *parS*c1 is less permissive than *parS* of single chromosomes. The analysis of nucleotide changes accepted in chromosomal *parS* have been carried out in *Bacillus subtilis* [15,35], *Streptomyces coelicolor* [36], *Pseudomonas aeruginosa* [34] and the main chromosome of *Vibrio cholerae—*which has two chromosomes [37]. Analysis were carried out by chromatin immunoprecipitation (ChIP), EMSA or fluorescence microscopy, testing only native *parS* variants, so that they are not as wide as our SPRi analysis. Nevertheless a single change at only one distal position and one single transition at position 5 or 12 (not the double change) naturally occurs in all *parS* and maintains an interaction with ParB, but less efficiently in chromosome 1 of *Vibrio cholerae*. To a lower extent, positions 7 or 10 appear permissive in *B*. *subtilis*, *P*. *aeruginosa* and *S*. *coelicolor*. By contrast in *V*. *cholerae*, like in *B*. *cenocepacia*, none of these latter positions are permissive to mutations. We cannot exclude from these results that the interaction between ParB and *parS* of the main chromosome is indeed more constrained in multi-chromosomal genomes.

As indicated, the single transition at position 5 or 12 naturally occurs on chromosomal *parS*, and the double transition at 5–12 maintains a high BS in our analysis. This well tolerated change could be finally fixed in some bacteria, allowing the emergence of a variant form of *parS*. In this direction, it is interesting to point out that, while in most species the canonical *parS* is TGTTTCACGTGAAACA—with only few repeats degenerated at position 5 or 1, all chromosomal *parSs* of *Pseudomonas* species share the motif TGTTCCACGTGGAACA [17,34], which is precisely changed at the position (5–12)—underlined. In *Pseudomonas* species the double symmetrical transition T5C-A12G certainly took over the original *parS* because of an undetermined selective pressure with a probable co-adaptation of ParB.

### Drawing evolving links between the other Par systems of *B*. *cenocepacia*

In *parS*c3, at least three positions (1–16, 2–15 and 6–11) are tolerant to changes (Fig 3B). We previously proposed that c3 has kept the features of an ancestral Par system from which 12D, 12J and G4 have emerged. The permissiveness of *parS*c3 to nucleotide changes strengthens this idea: like the position (5–12) of c1 discussed above, any nucleotide change accepted in the ancestral *parS*c3 might be fixed, evolving progressively a new *parS* sequence. The change G1A-C16T well accepted by *parS*c3, might have contributed to the formation of *parS*G4; the tolerance of *parS*c3 to the change C6A-G11T is less obvious—Fig 4B and Figure B in S1 File, but cannot be excluded. Further adapting modifications of ParBs would have allowed the emergence of new but related Par systems forming the Bsr3 family.

Beyond the evolutionary changes proposed within the Bsr3 family, other evolving links between the Par systems of *B*. *cenocepacia* could be considered, for example between pBC and c3. We recently found that the segregation of the origin of c3 is essentially mediated by its Par system, is less controlled by the cell cycle than those of c1 and c2, and is mostly similar to the segregation of pBC [38]. Thus c3 of *B*. *cenocepacia* could have a plasmid ancestor and this plasmid could be that of *B*. *cenocepacia* i.e. pBC. In this hypothesis, *parS*c3 might have formed from *parS*pBC. Three double changes are required in *parS*pBC for such a transformation: C1G-G16C, G5T-C12A and T7A-A10T. SPRi shows that the first change is very well accepted by *parS*pBC, but the two other ones much less (Fig 3C and Figure C in S1 File). Nevertheless among internal positions of *parS*pBC, (5–12) and (7–10) appear weakly tolerant to some changes. Besides once a mutation is fixed in a *parS* sequence, it generates a new *parS* motif, which should influence both an adaptation of ParB and the further capacity of *parS* to change again. That *parS*c3 form from *parS*pBC, even-though progressively, thus remains an attractive possibility.

## Supporting information

S1 File Sequences of all *parS* probes and binding scores obtained with the different ParB extracts. Each Figure in S1 File displays the data by ParB as follows: Figure A,ParBc1; Figure B, ParBc3; Figure C,ParBpBC; Figure D, ParB12D; Figure E,ParBG4; Figures F and G, ParB12J. Figures A-G in S1 File show for each probe: (i) the name (column “probe”); (ii) the 16bp sequence (column “sequence”), (iii) the mean BS and standard deviation with the extract over-producing ParB (column “BS ParB+”); (iv) the level of significance of the BS ParB+ (column “Sign”); (v) the mean BS and standard deviation with the extract non over-producing ParB (column “BS ParB-”,). BSs are presented as histograms to the right of each Figure.
(DOCX)
