# Supplementary material for: Analysis of ParB-centromere interactions by multiplex SPR imaging reveals specific patterns for binding ParB in six centromeres of Burkholderiales chromosomes and plasmids
Source: PLoS One. 2017 May 31;12(5):e0177056. doi: 10.1371/journal.pone.0177056 (PMC5450999; doi:10.1371/journal.pone.0177056)
Supplement: S1 File — Each Figure in S1 File displays the data by ParB as follows: Figure A,ParBc1; Figure B, ParBc3; Figure C,ParBpBC; Figure D, ParB12D; Figure E,ParBG4; Figures F and G, ParB12J. Figures A-G in S1 File show for each probe: (i) the name (column “probe”); (ii) the 16bp sequence (column “sequence”), (iii) the mean BS and standard deviation with the extract over-producing ParB (column “BS ParB+”); (iv) the level of significance of the BS ParB+ (column “Sign”); (v) the mean BS and standard deviation with the extract non over-producing ParB (column “BS ParB-”,). BSs are presented as histograms to the right of each Figure. (DOCX) [file pone.0177056.s001.docx]

**Figure A** (ParBc1)

**Probe^1^ sequence^2^ BS ParB+ Sign^3^ BS ParB-**

**S1wt TGTTTCACGTGAAACA 55 ± 10 ***** 10 ± 2

**S9half ATCTGATTGTGAAACA 27 ± 2 ***  3 ± 4

**S21rdm ATCTGATCCAGAATTG 9 ± 3**  8 ± 2

**S2 GGTTTCACGTGAAACA 47 ± 4 *****  7 ± 3

**S5 AGTTTCACGTGAAACA 40 ± 8 **** 12 ± 4

**S6 TGCTTCACGTGAAACA 43 ± 1 *****  9 ± 3

**S8 TGATTCACGTGAAACA 42 ± 2 ***** 16 ± 5

**S14 TGTATCACGTGAAACA 46 ± 3 ****  3 ± 3

**S16 TGTTACACGTGAAACA 43 ± 4 *****  8 ± 3

**S77 AGtttcacgtgaaaCg 36 ± 5 *****  3 ± 4

**S3 GGTTTCACGTGAAACC 39 ± 3 *****  6 ± 1

**S4 CGTTTCACGTGAAACC 41 ± 2 ****  4 ± 2

**S151  CGTTTCACGTGAAACG 43 ± 2 ***** 10 ± 3

**S10 AGTTTCACGTGAAACT 33 ± 4 ***  9 ± 4

**S11 TCTTTCACGTGAAAGA 9 ± 2**  2 ± 2

**
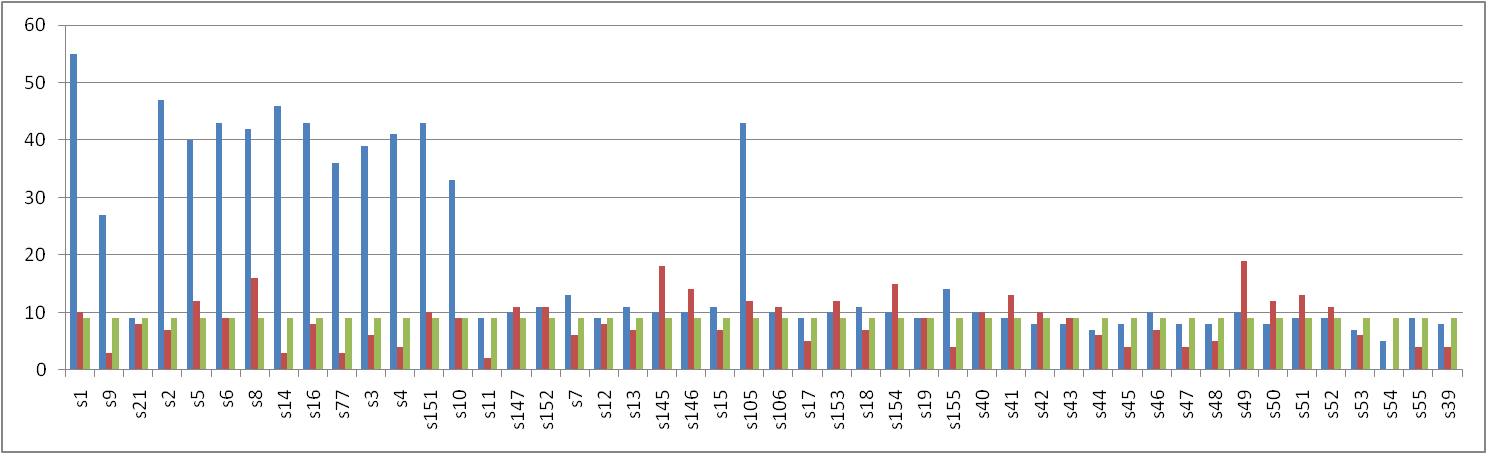
S147 TTTTTCACGTGAAAAA 10 ± 1** 11 ± 7

**S152  TATTTCACGTGAAATA 11 ± 1** 11 ± 5

**S7 TGCTTCACGTGAAGCA 13 ± 2**  6 ± 5

**S12 TGATTCACGTGAATCA 9 ± 2**  8 ± 3

**S13 TGTATCACGTGATACA 11 ± 1**  7 ± 3

**S145 TGTGTCACGTGACACA 10 ± 1** 18 ± 8

**S146 TGTCTCACGTGAGACA 10 ± 1** 14 ± 5

**S15 TGTTACACGTGTAACA 11 ± 1**  7 ± 6

**S105 TGTTCCACGTGGAACA 43 ± 5 ***** 12 ± 7

**S106 TGTTGCACGTGCAACA 10 ± 1** 11 ± 6

**S17 TGTTTGACGTCAAACA 9 ± 2**  5 ± 5

**S153  TGTTTTACGTAAAACA 10 ± 1** 12 ± 2

**S18 TGTTTCTCGAGAAACA 11 ± 1**  7 ± 2

**S154  TGTTTCGCGCGAAACA 10 ± 2**  15 ± 1

**S19 TGTTTCAGCTGAAACA 9 ± 1**  9 ± 4

**S155  TGTTTCATATGAAACA 14 ± 1**  4 ± 1

**S40 CttggctcgagccaaC 10 ± 2** 10 ± 4

**S41 GttggctcgagccaaC 9 ± 2** 13 ± 5

**S42 CAtggctcgagccaTG 8 ± 2** 10 ± 7

**S43 CtAggctcgagccTaG 8 ± 1**  9 ± 6

**S44 CttCgctcgagcGaaG 7 ± 1**  6 ± 2

**S45 CttgCctcgagGcaaG 8 ± 1**  4 ± 1

**S46 CttggGtcgaCccaaG 10 ± 2**  7 ± 6

**S47 CttggCtcgCGccaaG 8 ± 4**  4 ± 4

**S48 CttggCGcgCGccaaG 8 ± 2**  5 ± 3

**S49 CttggAtcgagccaaG 10 ± 1** 19 ± 5

**S50 CttggAtcgATccaaG 8 ± 1** 12 ± 2

**
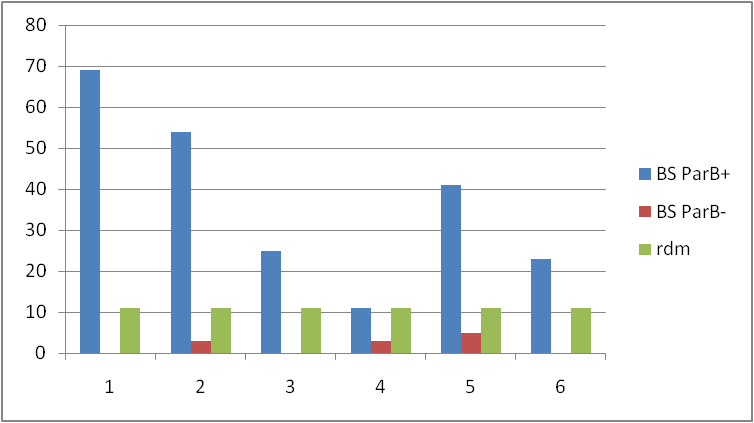
S51 CttggcAcgTgccaaG 9 ± 2** 13 ± 5

**S52 CttggctGCagccaaG 9 ± 2** 11 ± 3

**S53 CttggTtcgagccaaG 7 ± 1**  6 ± 3

**S54 CttggTtcgaAccaaG 5 ± 1**  0 ± 3

**S55 TGACGTGACGTGACGC 9 ± 3**  4 ± 5

**S39 TGACGTGTgagccaaG 8 ± 1**  4 ± 2

**Figure B** (ParBc3)

**Probe^1^ sequence^2^ BS ParB+ Sign^3^ BS ParB-**

**S56wt GttgtcacgtgacaaC 22 ± 7 **** 0 ± 3

**S57half TGACTGATgtgacaaC 17 ± 2 *** 0 ± 0

**S70rdm TGACTGACTGACTGAC 6 ± 4** 3 ± 3

**S58 TttgtcacgtgacaaC 26 ± 3 **** 0 ± 1

**S61 CttgtcacgtgacaaC 28 ± 11 **** 2 ± 1

**S74 GttgtTacgtGacaaC 28 ± 1 **** 3 ± 2

**S107 ATTGTCACGTGACAAT 21 ± 7 **** 3 ± 4

**S59 TttgtcacgtgacaaA 24 ± 2 * 3 ± 2**

**S60 AttgtcacgtgacaaA 20 ± 8 **** 2 ± 1

**S62 CttgtcacgtgacaaG 10 ± 3** 6 ± 4

**S63 GAtgtcacgtgacaTC 16 ± 2 **** 2 ± 2

**S108 GCTGTCACGTGACAGC 12 ± 3** 4 ± 3

**S109 GTCGTCACGTGACGAC 5 ± 2** 2 ± 3

**S110 GTGGTCACGTGACCAC 10 ± 2** 2 ± 1

**S64 GtAgtcacgtgacTaC 16 ± 5** 2 ± 4

**S65 GttCtcacgtgaGaaC 11 ± 6** 2 ± 2

**S71 GttTtcacgtgaAaaC 10 ± 2** 3 ± 1

**
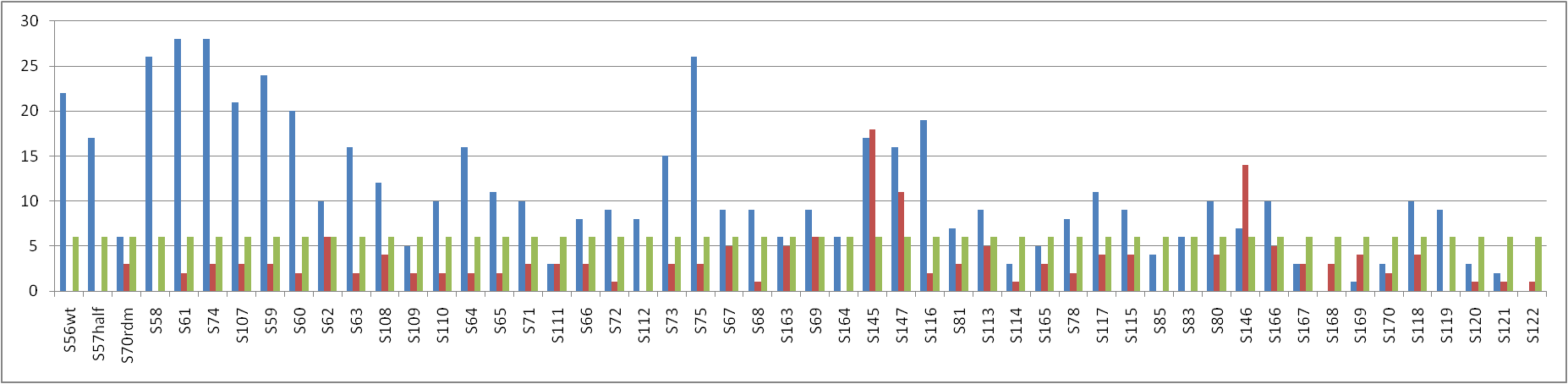
S111 GTTATCACGTGATAAC 3 ± 5** 3 ± 2

**S66 GttgAcacgtgTcaaC 8 ± 4** 3 ± 2

**S72 GttgCcacgtgGcaaC 9 ± 2** 1 ± 1

**S112 GTTGGCACGTGCCAAC 8 ± 4** na

**S73 GttgtAacgtTacaaC 15 ± 3** 3 **±** 3

**S75 GttgtTacgtAacaaC 26 ± 5 **** 3 **±** 1

**S67 GttgtGacgtCacaaC 9 ± 4** 5 **±** 1

**S68 GttgtcTcgAgacaaC 9 ± 8** 1 **±** 0

**S163 GttgtcGcgCgacaaC 6 ± 8** 5 **±** 2

**S69 GttgtcaGCtgacaaC 9 ± 6** 6 **±** 6

**S164 GttgtcaTAtgacaaC 6 ± 3** 0 ± 2

**S145 TGTGTCACGTGACACA 17 ± 2** 18 **±** 8

**S147 TTTTTCACGTGAAAAA 16 ± 3** 11 ± 7

**S116 GTTTTAACGTTAAAAC 19 ± 2** 2 ± 2

**S81 GTTgCaacgttgCaac 7 ± 2** 3 ± 1

**S113 GTTTCCACGTGGAAAC 9 ± 6** 5 ± 1

**S114 GTTGCTACGTAGCAAC 3 ± 2** 1 ± 1

**S165 GttTtTacgtAaAaaC 5 ± 6** 3 ± 6

**S78 GTTTCaacgttgaaac 8 ± 5** 2 ± 2

**S117 GTTTAAACGTTTAAAC 11 ± 2** 4 ± 2

**S115 GTTTGAACGTTCAAAC 9 ± 2** 4 ± 2

**S85 gtttctacgtagaaac 4 ± 6** 0 ± 3

**S83 GTTCCaacgttgGaac 6 ± 4** 0 ± 3

**S80 GTTTCGacgtCgaaac 10 ± 5** 4 ± 1

**S146 TGTCTCACGTGAGACA 7 ± 3** 14 ± 5

**S166 ATTTCaacgttgaaaT 10 ± 5** 5 ± 2

**S167 GCTTCaacgttgaaGc 3 ± 3** 3 ± 3

**S168 GTCTCaacgttgaGac 0 ± 5** 3 ± 3

**S169 GTTTCaGcgCtgaaac 1 ± 3** 4 ± 3

**
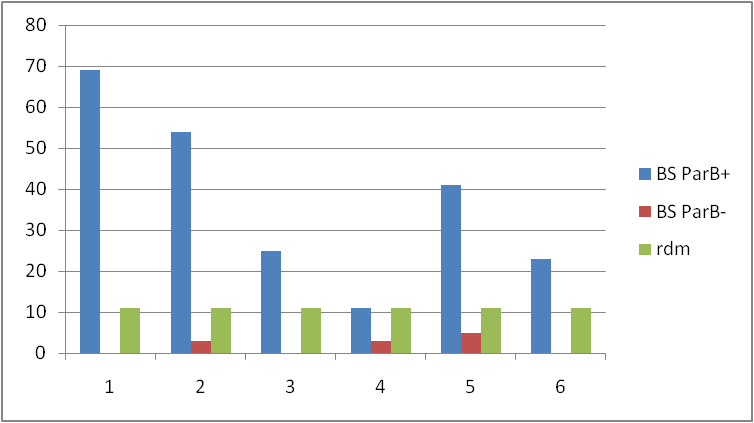
S170 GTTTCaaTAttgaaac 3 ± 3** 2 ± 1

**S118 CTTTCaacgttgaaaG 10 ± 4** 4 **±** 2

**S119 GATTCaacgttgaaTc 9 ± 4** 0 **±** 3

**S120 GTATCaacgttgaTac 3 ± 5** 1 **±** 3

**S121 GTTTCaTcgAtgaaac 2 ± 7** 1 **±** 4

**S122 GTTTCaaGCttgaaac 0 ± 1** 1 **±** 1

**Figure C** (ParBpBC)

**Probe^1^ sequence^2^ BS ParB+ Sign^3^ BS ParB-**

**
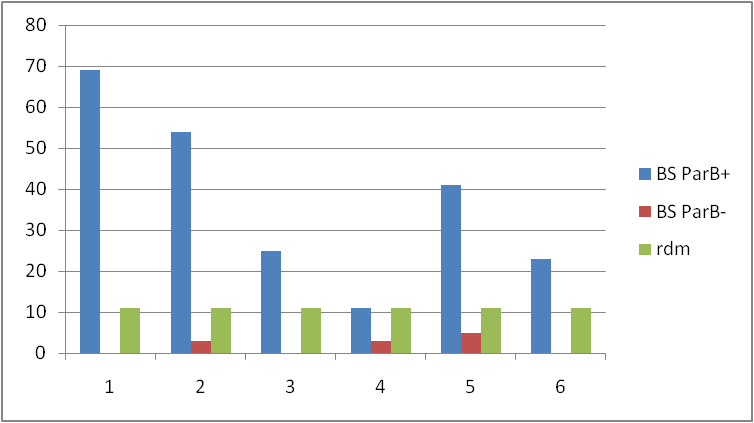

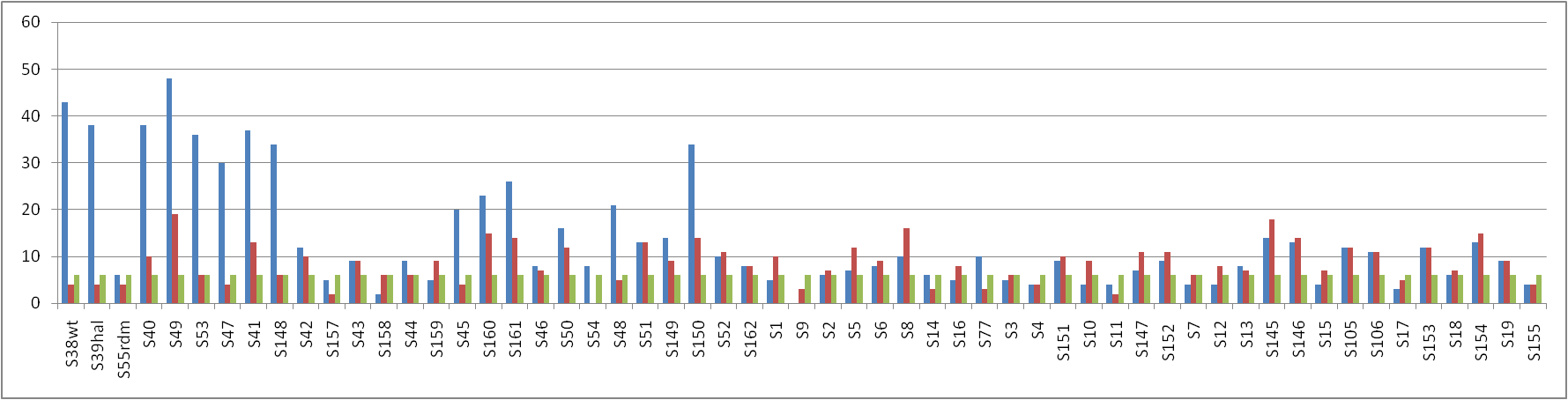
S38wt CttggctcgagccaaG 43 ± 4 *****  4 ± 0 **S39half TGACGTGTgagccaaG 38 ± 7 ****  4 ± 2 **S55rdm TGACGTGACGTGACGC 6 ± 1**  4 ± 5  **S40 GttggctcgagccaaG 38 ± 3 ***** 10 ± 4 **S49 CttggAtcgagccaaG 48 ± 5 **** 19 ± 5 **S53 CttggTtcgagccaaG 36 ± 4 ****  6 ± 3 **S47 CttggCGcgAGccaaG 30 ± 1 *****  4 ± 4 **S41 GttggctcgagccaaC 37 ± 5 ***** 13 ± 5 **S148 ATTGGCTCGAGCCAAT 34 ± 1 *****  6 ± 4 **S42 CAtggctcgagccaTG 12 ± 3** 10 ± 7 **S157 CCtggctcgagccaGG 5 ± 3**  2 ± 4 **S43 CtAggctcgagccTaG 9 ± 5**  9 ± 6  **S158 CtCggctcgagccGaG 2 ± 2**  6 ± 1 **S44 CttCgctcgagcGaaG 9 ± 2**  6 ± 2 **S159 CttAgctcgagcTaaG 5 ± 1**  9 ± 4 **S45 CttgCctcgagGcaaG 20 ± 5**  4 ± 1 **S160 CttgActcgagTcaaG 23 ± 2** 15 ± 4  **S161 CttgTctcgagAcaaG 26 ± 1** 14 ± 5 **S46 CttggGtcgaCccaaG 8 ± 6**  7 ± 6 **S50 CttggAtcgATccaaG 16 ± 1** 12 ± 2 **S54 CttggTtcgaAccaaG 8 ± 3**  0 ± 3 **S48 CttggCGcgCGccaaG 21 ± 6 ***  5 ± 3 **S51 CttggcAcgTgccaaG 13 ± 1** 13 ± 5 **S149 CTTGGCGCGTGCCAAG 14 ± 1**  9 ± 3 **S150 CTTGGCCCGGGCCAAG 34 ± 7 **** 14 ± 9 **S52 CttggctGCagccaaG 10 ± 2** 11 ± 3 **S162 CttggctTAagccaaG 8 ± 1**  8 ± 4 **S1 TGTTTCACGTGAAACA 5 ± 3** 10 ± 2 **S9 ATCTGATTGTGAAACA 0 ± 2**  3 ± 4 **S2 TGTTTCACGTGAAACC 6 ± 2**  7 ± 3 **S5 TGTTTCACGTGAAACT 7 ± 1** 12 ± 5 **S6 TGCTTCACGTGAAACA 8 ± 3**  9 ± 3 **S8 TGATTCACGTGAAACA 10 ± 1** 16 ± 5 **S14 TGTATCACGTGAAACA 6 ± 3**  3 ± 3 **S16 TGTTACACGTGAAACA 5 ± 2**  8 ± 3 **S77 AGtttcacgtgaaaCg 10 ± 2**  3 ± 4 **S3 GGTTTCACGTGAAACC 5 ± 1**  6 ± 1 **S4 CGTTTCACGTGAAACC 4 ± 3**  4 ± 2 **S151  CGTTTCACGTGAAACG 9 ± 1** 10 ± 3 **S10 AGTTTCACGTGAAACT 4 ± 4**  9 ± 4 **S11 TCTTTCACGTGAAAGA 4 ± 1**  2 ± 2 **S147 TTTTTCACGTGAAAAA 7 ± 2** 11 ± 7 **S152  TATTTCACGTGAAATA 9 ± 3** 11 ± 5 **S7 TGCTTCACGTGAAGCA 4 ± 3**  6 ± 5 **S12 TGATTCACGTGAATCA 4 ± 3**  8 ± 3 **S13 TGTATCACGTGATACA 8 ± 2**  7 ± 3 **S145 TGTGTCACGTGACACA 14 ± 5** 18 ± 8 **S146 TGTCTCACGTGAGACA 13 ± 2** 14 ± 5 **S15 TGTTACACGTGTAACA 4 ± 3**  7 ± 6 **S105 TGTTCCACGTGGAACA 12 ± 4** 12 ± 7 **S106 TGTTGCACGTGCAACA 11 ± 4** 11 ± 6 **S17 TGTTTGACGTCAAACA 3 ± 3**  5 ± 5 **S153  TGTTTTACGTAAAACA 12 ± 1** 12 ± 2 **S18 TGTTTCTCGAGAAACA 6 ± 2**  7 ± 2 **S154  TGTTTCGCGCGAAACA 13 ± 1** 15 ± 1 **S19 TGTTTCAGCTGAAACA 9 ± 3**  9 ± 4 **S155  TGTTTCATATGAAACA 4 ± 1**  4 ± 1

**Figure D** (ParB12D)

**probe^1^ sequence^2^ BS ParB+ Sign^3^ BS ParB-**

**S78wt GTTTCaacgttgaaac 42 ± 5 ***** 2 ± 2

**S179half TAACTGATgttgaaac 12 ± 3** 1 ± 3

**S70rdm TGACTGACTGACTGAC 3 ± 4** 3 ± 3

**S118 CTTTCaacgttgaaaG 18 ± 3** 4 ± 2

**S166 ATTTCaacgttgaaaT 28 ± 6 *** 5 ± 2
**S119 GATTCaacgttgaaTc 6 ± 3** 0 ± 3

**S167 GCTTCaacgttgaaGc 8 ± 2** 3 ± 3

**S120 GTATCaacgttgaTac 3 ± 3** 1 ± 3

**S168 GTCTCaacgttgaGac 6 ± 4** 3 ± 3

**S82 GTTACaacgttgTaac 7 ± 5** 2 ± 1

**S81 GTTgCaacgttgCaac 9 ± 3** 3 ± 1

**S83 GTTCCaacgttgGaac 5 ± 3** 0 ± 3

**S115 GTTTGAACGTTCAAAC 9 ± 3** 4 ± 2

**S116 GTTTTAACGTTAAAAC 8 ± 1** 2 ± 2

**S117 GTTTAAACGTTTAAAC 8 ± 1** 4 ± 2

**S79 GTTTCCacgtGgaaac 17 ± 8** 5 ± 1

**S80 GTTTCGacgtCgaaac 11 ± 3** 4 ± 1

**S85 gtttctacgtagaaac 5 ± 6** 0 ± 3

**S121 GTTTCaTcgAtgaaac 12 ± 6** 1 ± 4

**S169 GTTTCaGcgCtgaaac 16 ± 1** 4 ± 3

**
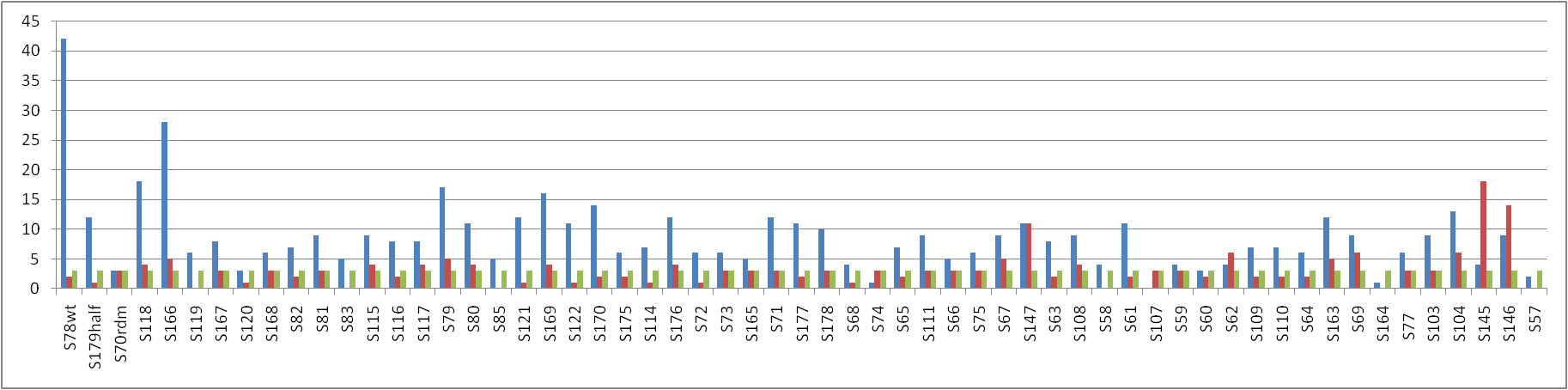
S122 GTTTCaaGCttgaaac 11 ± 5** 1 ± 1

**S170 GTTTCaaTAttgaaac 14 ± 1** 2 ± 1

**S175 GCTTCCACgtggaaGc 6 ± 1** 2 ± 1

**S114 GTTGCTACGTAGCAAC 7 ± 4** 1 ± 1

**S176 GTTCCCACgtggGaac 12 ± 4** 4 ± 1

**S72 GttgCcacgtgGcaaC 6 ± 3** 1 ± 1

**S73 GttgtAacgtTacaaC 6 ± 4** 3 ± 3

**S165 GttTtTacgtAaAaaC 5 ± 4** 3 ± 6

**S71 GttTtcacgtgaAaaC 12 ± 5** 3 ± 1

**S177 GTTTCCGCgCggaaac 11 ± 3** 2 ± 5

**S178 GTTTCCATAtggaaac 10 ± 5** 3 ± 3

**S68 GttgtcTcgAgacaaC 4 ± 1** 1 ± 0

**S74 GttgtTacgtGacaaC 1 ± 2** 3 ± 2

**S65 GttCtcacgtgaGaaC 7 ± 3** 2 ± 2

**S111 GTTATCACGTGATAAC 9 ± 3** 3 ± 2

**S66 GttgAcacgtgTcaaC 5 ± 5** 3 ± 2

**S75 GttgtTacgtAacaaC 6 ± 3** 3 ± 1

**S67 GttgtGacgtCacaaC 9 ± 3** 5 ± 1

**S147 TTTTTCACGTGAAAAA 11 ± 7** 11 ± 7

**S63 GAtgtcacgtgacaTC 8 ± 4** 2 ± 2

**S108 GCTGTCACGTGACAGC 9 ± 6** 4 ± 3

**S58 GttgtcacgtgacaaA 4 ± 2** 0 ± 1

**S61 GttgtcacgtgacaaG 11 ± 7** 2 ± 1

**S107 ATTGTCACGTGACAAT 0 ± 2** 3 ± 4

**S59 TttgtcacgtgacaaA 4 ± 4** 3 ± 2

**S60 AttgtcacgtgacaaA 3 ± 2** 2 ± 1

**S62 CttgtcacgtgacaaG 4 ±7** 6 ± 4

**S109 GTCGTCACGTGACGAC 7 ± 7** 2 ± 3

**S110 GTGGTCACGTGACCAC 7 ± 1** 2 ± 1

**S64 GtAgtcacgtgacTaC 6 ± 6** 2 ± 4

**S163 GttgtcGcgCgacaaC 12 ± 6** 5 ± 2

**S69 GttgtcaGCtgacaaC 9 ± 7** 6 ± 6

**
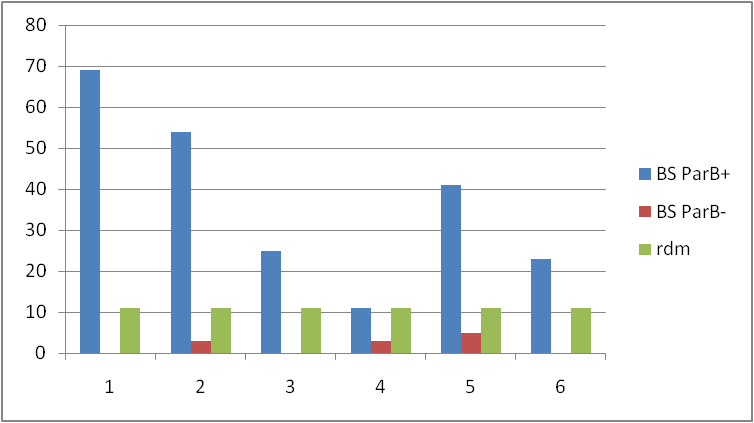
S164 GttgtcaTAtgacaaC 1 ± 3** 0 ± 2

**S77 AGtttcacgtgaaaCg 6 ± 3** 3 ± 4

**S103 CTTTCACCGGTGAAAG 9 ± 2** 3 ± 2

**S104 GTTCAACCGGTTGAAC 13 ± 4** 6 ± 1

**S145 TGTGTCACGTGACACA 4 ± 1** 18 ± 8

**S146 TGTCTCACGTGAGACA 9 ± 3** 14 ± 5

**S57 TGACTGATgtgacaaC 2 ± 3** 0 ± 0

**Figure E** (ParBG4)

**Probe^1^ sequence^2^ BS ParB+ Sign^3^ BS ParB-**

**S89wt ATCTccacgtggagat 63 ± 6 **** 0 ± 3

**S181half TCACCTCAgtggagat 24 ± 3** na

**S70rdm TGACTGACTGACTGAC 27 ± 9** 3 ± 3

**S99 ATCGTcacgtggagat 38 ± 3** 6 ± 0

**S98 ATTGccacgtggagat 36 ± 5** 3 ± 3

**
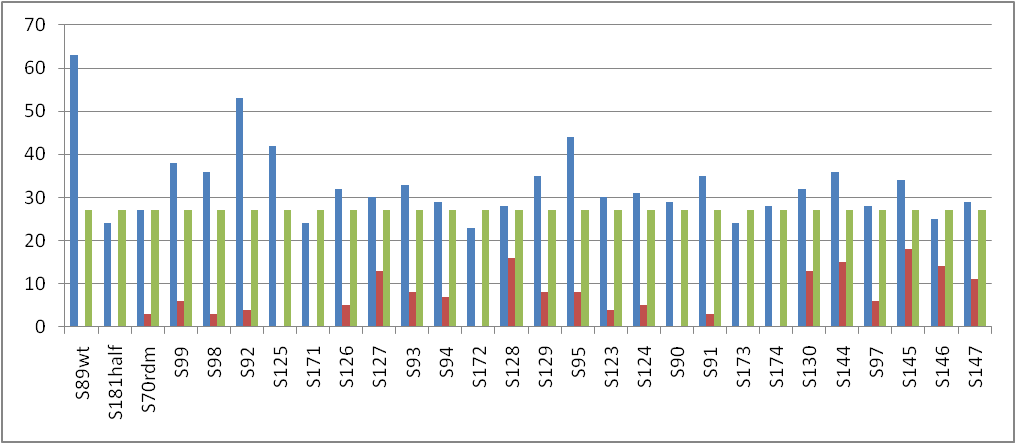
S92 GTCTccacgtggagaC 53 ± 4 **** 4 ± 2

**S125 TTCTccacgtggagaA 42 ± 2 *** 0 ± 1

**S171 ACCTccacgtggagGt 24 ± 6** na

**S126 AACTccacgtggagTt 32 ± 1** 5 ± 2

**S127 ATGTccacgtggaCat 30 ± 2** 13 ± 1

**S93 ATTTccacgtggaAat 33 ± 4** 8 ± 2

**S94 ATCGccacgtggCgat 29 ± 3** 7 ± 0

**S172 ATCCccacgtggGgat 23 ± 5** na

**S128 ATCAccacgtggTgat 28 ± 7** 16 ± 3

**S129 ATCTGcacgtgCagat 35 ± 7** 8 ± 4

**S95 ATCTTcacgtgAagat 44 ± 6 *** 8 ± 2

**S123 ATCTCTACGTAGAGAT 30 ± 4** 4 ± 1

**S124 ATCTCGACGTCGAGAT 31 ± 5** 5 ± 2

**S90 ATCTcAacgtTgagat 29 ± 1** 0 ± 4

**S91 ATCTccTcgAggagat 35 ± 2** 3 ± 2

**S173 ATCTccGcgCggagat 24 ± 6** na

**S174 ATCTccaTAtggagat 28 ± 2** na

**
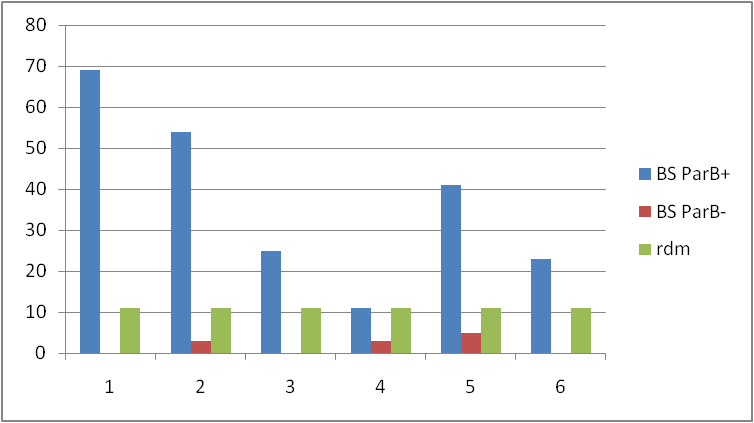
S130 ATCTccaGCtggagat 32 ± 3** 13 ± 4

**S144 GTTTCCAGCtggaaac 36 ± 2** 15 ± 5

**S97 GTCGccacgtggCgaC 28 ± 2** 6 ± 2

**S145 TGTGTCACGTGACACA 34 ± 6** 18 ± 8

**S146 TGTCTCACGTGAGACA 25 ± 5** 14 ± 5

**S147 TTTTTCACGTGAAAAA 29 ± 4** 11 ± 7

**Figure F (**ParB12J; changes relative to probe S56, *i.e*. *parS*c3)

**probe^1^ sequence^2^ BS ParB+ Sign^3^ BS ParB-**

**S56 GttgtcacgtgacaaC 69 ± 8 *** 0 ± 3

**S86wt GTTgTcacgtggaaac 54 ± 22 *** 3 ± 0

**S57half TGACTGATgtgacaaC 25 ± 22** 0 ± 0

**S70rdm TGACTGACTGACTGAC 11 ± 11** 3 ± 3

**S88 GTTTCCACgtggaaac 41 ± 17** 5 ± 1

**S58 GttgtcacgtgacaaA 23 ± 21** 0 ± 1

**S61 GttgtcacgtgacaaG 20 ± 15** 2 ± 1

**S74 GttgtTacgtGacaaC 37 ± 22** 3 ± 2

**S59 TttgtcacgtgacaaA 22 ± 21** 3 ± 2

**S60 AttgtcacgtgacaaA 24 ± 13** 2 ± 1

**S62 CttgtcacgtgacaaG 21 ± 13** 6 ± 4

**S63 GAtgtcacgtgacaTC 21 ± 14** 2 ± 2

**
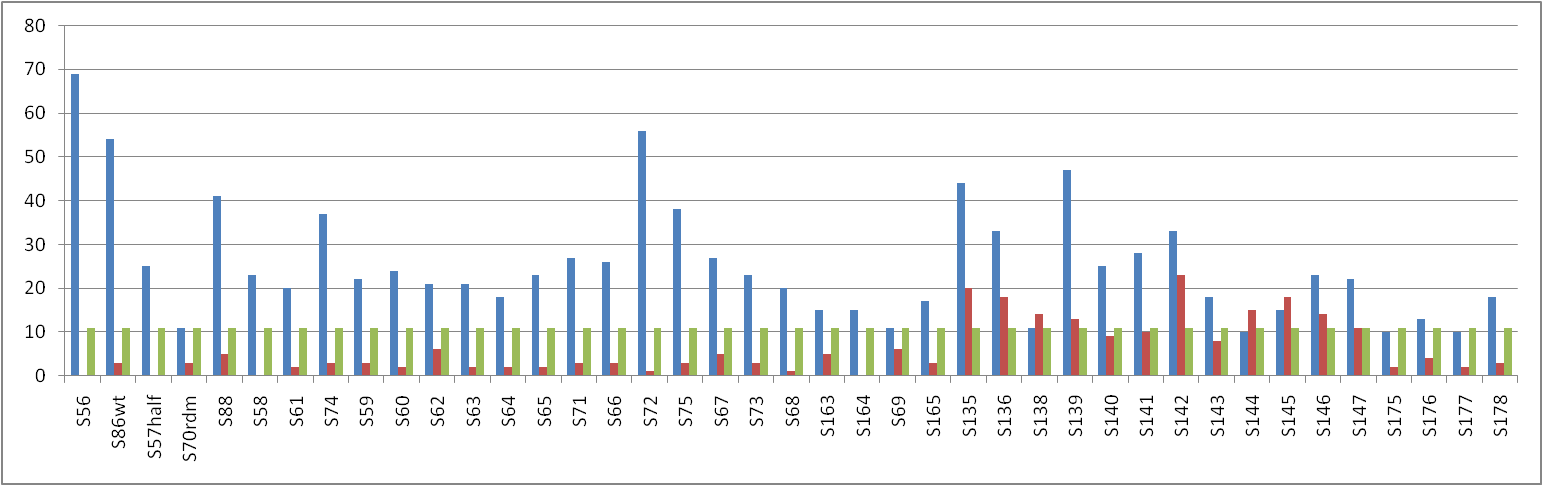

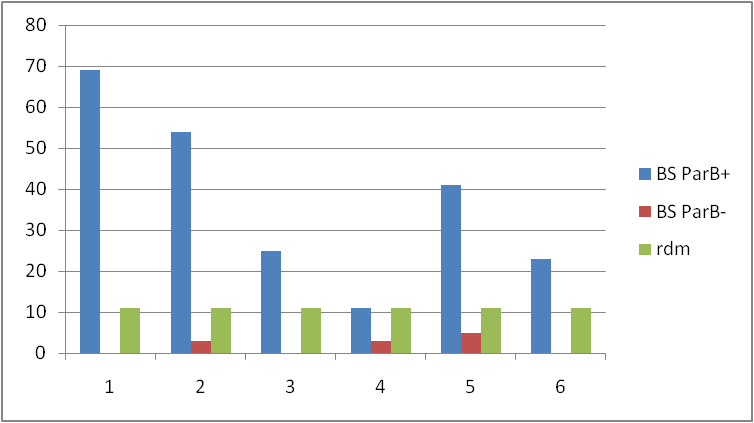
S64 GtAgtcacgtgacTaC 18 ± 12** 2 ± 4

**S65 GttCtcacgtgaGaaC 23 ± 16** 2 ± 2

**S71 GttTtcacgtgaAaaC 27 ± 21** 3 ± 1

**S66 GttgAcacgtgTcaaC 26 ± 15** 3 ± 2

**S72 GttgCcacgtgGcaaC 56 ± 23 *** 1 ± 1

**S75 GttgtTacgtAacaaC 38 ± 30** 3 ± 1

**S67 GttgtGacgtCacaaC 27 ± 17** 5 ± 1

**S73 GttgtAacgtTacaaC 23 ± 15** 3 ± 3

**S68 GttgtcTcgAgacaaC 20 ± 17** 1 ± 0

**S163 GttgtcGcgCgacaaC 15 ± 8** 5 ± 2

**S164 GttgtcaTAtgacaaC 15 ± 3** 0 ± 2

**S69 GttgtcaGCtgacaaC 11 ± 1** 6 ± 6

**S165 GttTtTacgtAaAaaC 17 ± 5** 3 ± 6

**S135 GTTGCCACGTGGAAAC 44 ± 23** 20 ± 2

**S136 GTTTTCACGTGGAAAC 33 ± 23** 18 ± 1

**S138 CTTTCCACgtggaaaG 11 ± 8** 14 ± 0

**S139 GATTCCACgtggaaTc 47 ± 31 *** 13 ± 1

**S140 GTATCCACgtggaTac 25 ± 15** 9 ± 1

**S141 GTTACCACgtggTaac 28 ± 11 *** 10 ± 4

**S142 GTTTGCACgtgCaaac 33 ± 7** 23 ± 5

**S143 GTTTCCTCgAggaaac 18 ± 8** 8 ± 2

**S144 GTTTCCAGCtggaaac 10 ± 5** 15 ± 5

**S145 TGTGTCACGTGACACA 15 ± 8** 18 ± 8

**S146 TGTCTCACGTGAGACA 23 ± 8** 14 ± 5

**
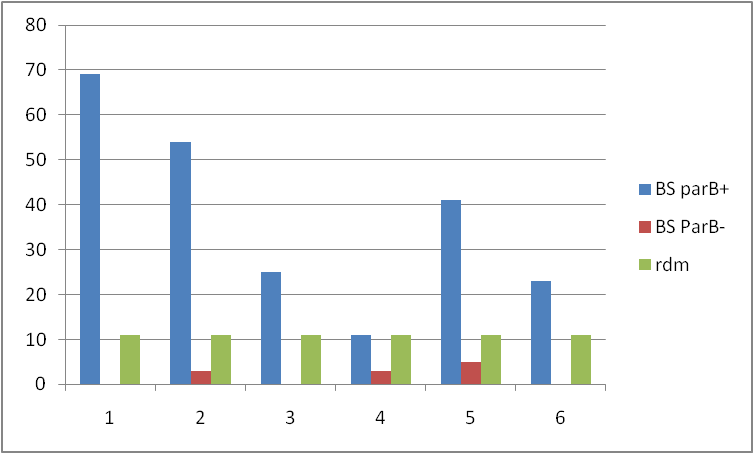
S147 TTTTTCACGTGAAAAA 22 ± 15** 11 ± 7

**S175 GCTTCCACgtggaaGc 10 ± 5** 2 ± 1

**S176 GTTCCCACgtggGaac 13 ± 6** 4 ± 1

**S177 GTTTCCGCgCggaaac 10 ± 7** 2 ± 5

**S178 GTTTCCATAtggaaac 18 ± 10** 3 ± 3

**Figure G** (ParB12J ; changes relative to probe S88, *i.e.* “non-*parS*c3”)

**Probe^1^ sequence^2^ BS ParB+ Sign^3^ BS ParB-**

**S88 GTTTCCACgtggaaac 41 ± 17**  5 ± 1

**S86wt gTTgTcacgtggaaac 54 ± 22 *** 3 ± 0

**S180halfTACCTGCTgtggaaac 14 ± 4** na

**S70rdm TGACTGACTGACTGAC 11 ± 11** 3 ± 3

**S56 GttgtcacgtgacaaC 69 ± 8 *** 0 ± 3

**S136 GTTTTCACGTGGAAAC 33 ± 23** 18 ± 1

**S135 GTTGCCACGTGGAAAC 44 ± 23** 20 ± 2

**S143 GTTTCCTCgAggaaac 18 ± 8** 8 ± 2

**S138 CTTTCCACgtggaaaG 11 ± 8** 14 ± 0

**S175 GCTTCCACgtggaaGc 10 ± 5** 2 ± 1

**S139 GATTCCACgtggaaTc 47 ± 31 *** 13 ± 1

**S140 GTATCCACgtggaTac 25 ± 15** 9 ± 1

**
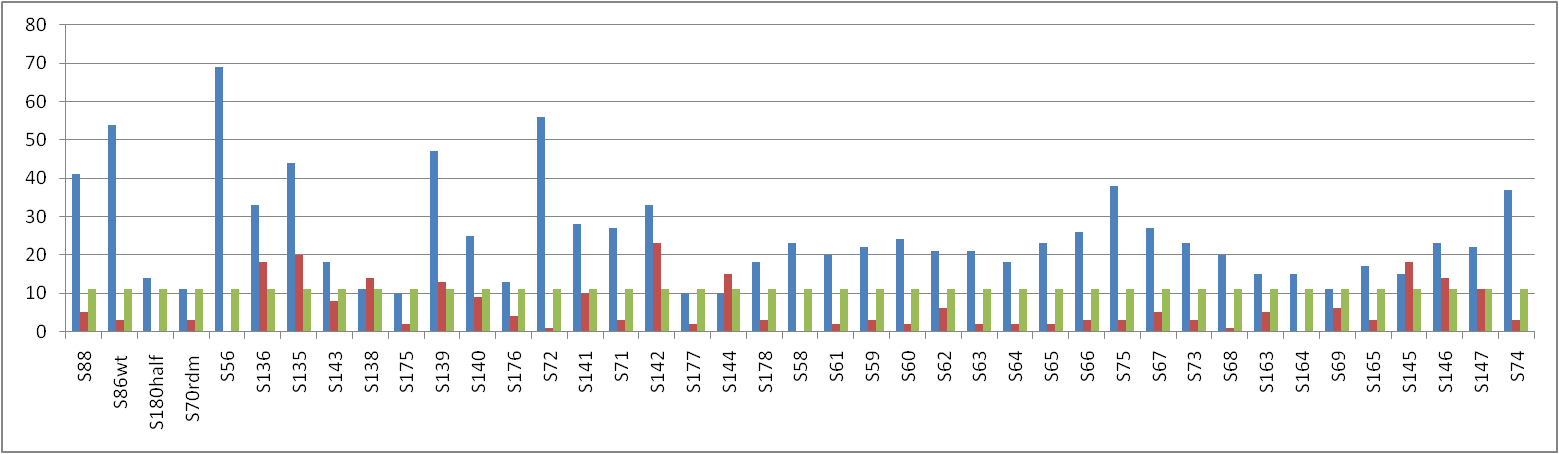
S176 GTTCCCACgtggGaac 13 ± 6** 4 ± 1

**S72 GTTGCCACGTGGCAAC 56 ± 23 *** 1 ± 1

**S141 GTTACCACgtggTaac 28 ± 11 *** 10 ± 4

**S71 GttTtcacgtgaAaaC 27 ± 21** 3 ± 1

**S142 GTTTGCACgtgCaaac 33 ± 7** 23 ± 5

**S177 GTTTCCGCgCggaaac 10 ± 7** 2 ± 5

**S144 GTTTCCAGCtggaaac 10 ± 5** 15 ± 5

**S178 GTTTCCATAtggaaac 18 ± 10** 3 ± 3

**S58 GttgtcacgtgacaaA 23 ± 21** 0 ± 1

**S61 GttgtcacgtgacaaG 20 ± 15** 2 ± 1

**S59 TttgtcacgtgacaaA 22 ± 21** 3 ± 2

**S60 AttgtcacgtgacaaA 24 ± 13** 2 ± 1

**S62 CttgtcacgtgacaaG 21 ± 13** 6 ± 4

**S63 GAtgtcacgtgacaTC 21 ± 14** 2 ± 2

**S64 GtAgtcacgtgacTaC 18 ± 12** 2 ± 4

**S65 GttCtcacgtgaGaaC 23 ± 16** 2 ± 2

**S66 GttgAcacgtgTcaaC 26 ± 15** 3 ± 2

**S75 GttgtTacgtAacaaC 38 ± 30** 3 ± 1

**S67 GttgtGacgtCacaaC 27 ± 17** 5 ± 1

**S73 GttgtAacgtTacaaC 23 ± 15** 3 ± 3

**S68 GttgtcTcgAgacaaC 20 ± 17** 1 ± 0

**S163 GttgtcGcgCgacaaC 15 ± 8** 5 ± 2

**S164 GttgtcaTAtgacaaC 15 ± 3** 0 ± 2

**
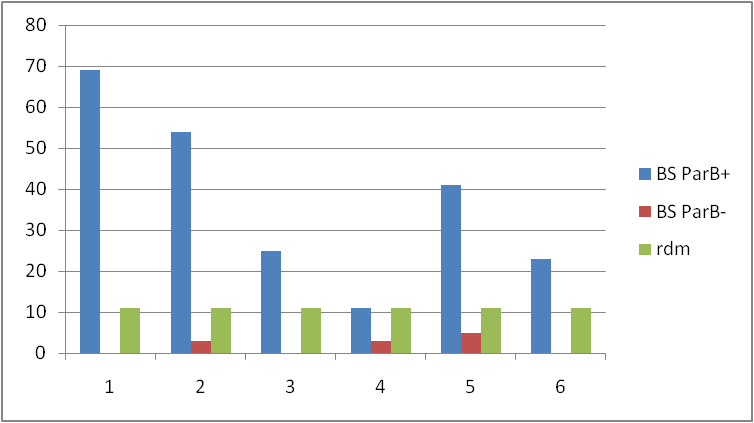
S69 GttgtcaGCtgacaaC 11 ± 10** 6 ± 6

**S165 GttTtTacgtAaAaaC 17 ± 5** 3 ± 6

**S145 TGTGTCACGTGACACA 15 ± 8** 18 ± 8

**S146 TGTCTCACGTGAGACA 23 ± 8** 14 ± 5

**S147 TTTTTCACGTGAAAAA 22 ± 15** 11 ± 7

**S74 GttgtTacgtGacaaC 37 ± 22** 3 ± 2

^1^ Wild-type (wt), random (rdm) and half-*parS* (half) sequences are mentioned for clarity

^2^ Red-highlited nucleotides in the *parS* sequences are those changed relative to the cognate wt *parS* (Figures A-E) or to *parS*c3 (Figure F) or to “non-*parS*c3” (Figure G)

^3^ Levels of significance are indicated in the “Sign” columns as follows:
* (P value < 0.05), ** (P value < 0.01), *** (P value <0.001).
